# Fecal calprotectin levels are higher in rural than in urban Chinese infants and negatively associated with growth

**DOI:** 10.1186/1471-2431-12-129

**Published:** 2012-08-23

**Authors:** Jin-Rong Liu, Xiao-Yang Sheng, Yan-Qi Hu, Xiao-Gang Yu, Jamie E Westcott, Leland V Miller, Nancy F Krebs, K Michael Hambidge

**Affiliations:** 1 Department of Child and Adolescent Health Care, MOE-Shanghai Key Laboratory of Children’s Environmental Health, Xinhua Hospital, Shanghai Jiao Tong University, School of Medicine, Shanghai Key Laboratory of Pediatric Gastroenterology and Nutrition, Shanghai Institute for Pediatric Research, 1665 Kongjiang Road, Shanghai, 200092, China; 2 University of Colorado School of Medicine, Department of Pediatrics, Section of Nutrition, Box C225, Research Complex II, 12700 East 19th Avenue, Aurora, CO 80045, USA

**Keywords:** Fecal calprotectin, Infants, Children, Gut inflammation, Growth

## Abstract

**Background:**

Fecal calprotectin (FC) is an established simple biomarker of gut inflammation. To examine a possible relationship between linear growth and gut inflammation, we compared fecal calprotectin levels in 6 month old infants from poor rural vs affluent urban families.

**Methods:**

The project was a cross-sectional comparison of FC from rural and urban populations in China. The relationship between length-for-age Z-score (LAZ) and FC concentrations were also compared. Single fecal samples were assayed for FC using EK-CAL ELISA kits.

**Results:**

The age of subjects for both locations was 6.1 ± 0.2 mo; all were apparently healthy. The mean ± SD of the LAZ for the rural and urban infants were −0.6 ± 0.9 and 0.4 ± 0.9, respectively. FC had a non-normal distribution. The median FC of 420.9 and 140.1 μg/g for rural and urban infants, respectively, were significantly different (*P* < 0.0001). For the rural group, linear regression analysis showed that an increase in FC of 100 μg/g was associated with a decrease of 0.06 in LAZ.

**Conclusion:**

FC levels were significantly elevated in the rural infants and high concentrations accounted for approximately one-third of the low LAZ scores of these infants.

## Background

The unhygienic and unsanitary environment typical of so many children in poor communities worldwide has been incriminated as a factor of note in the early onset of impaired linear growth in infants in these communities [1]. However, quantitative information remains limited on the role of unhygienic, unsanitary, poor environments per se on intestinal inflammation and associated linear growth failure [2-5]. Lunn and Campbell, et al. [2] proposed that frequent gastrointestinal infections, arising from unhygienic and unsanitary environments, impair small intestinal mucosal function, and explained up to 64% of the observed height and weight faltering of Gambian infants. Recently, works in Bangladesh and Nepal further confirmed the association of gut damage and growth faltering [6,7].

Calprotectin is a calcium and zinc binding protein in the S100 family with a total molecular mass of 36.5 kD [8-10]. It is abundant in neutrophilic granulocytes, monocytes and macrophages, accounting for 60% of all cytosolic protein in neutrophils. Neutrophils comprise part of the intestinal innate immune system and pass through the intestinal wall resulting in increase in secretion of calprotectin which is excreted in the feces intact. Fecal calprotectin (FC) is an established biomarker of intestinal inflammation [11-14]. Most recently, fecal calprotectin levels have also been found to be associated with persistent Giardia and microscopic duodenal inflammation [15]. Only a gram or less of sample is required with improved assays and the FC is remarkably resilient to degradation for more than a week at room temperature [16]. The application of FC assays provides a promising opportunity for comparison of the occurrence and severity of intestinal inflammation between communities exposed to different environments. We hypothesized that FC levels in mid-infancy are higher in a rural population than in a middle income urban population, and that FC levels are negatively associated with the rural infants’ growth.

## Methods

### Study design

The project was a cross-sectional comparison of fecal calprotectin concentrations in infants at the age of 6 mo in two Chinese populations: one poor rural, the other upper-middle income urban. We also examined the relationship between length-for-age Z-scores (LAZ) and fecal calprotectin levels.

### Subjects

The subjects were infants aged 6 ± 1 mo. One-hundred-and-five infants were located in the relatively poor rural county of Xichou located in southwest China. All were apparently healthy with no evidence of acute or chronic disease or congenital malformations. This study resulted from a hypothesis that developed late in the course of 6-mo baseline data collection for the primary study, and rural samples were limited to all infants - no refusals or exclusions and none with recent history of diarrhea - who had not already had their baseline visit. Hence sample size was dictated by practical limitations rather than by power testing. The primary study was a complementary feeding study commencing after these baseline studies (Sheng et at, unpublished). The rural subjects were from 60 rural villages in Xichou county. The sample size for the urban infants was also dictated by practical considerations. For a limited time period all infants seen at Kongjiang Community Health Center, Shanghai, for a routine health check at age 6 months were invited to participate with 100% acceptance. These were healthy term singleton infants from upper-middle class families delivered between 37–42 wk gestation with birth weights of 2,500-4000 g. Infants with history of diarrhea or respiratory tract infection within previous 2 weeks were excluded.

Written informed consent was obtained from the mother of each infant. The research protocol was approved by Shanghai Jiao Tong University School of Medicine Xinhua Hospital Institute Review Board and the Colorado Multiple Institutional Review Board.

### Anthropometric measurements

Infant anthropometric measurements were undertaken in duplicate by a trained member of the research team. Lengths were measured following standard techniques using a portable seca infantometer (seca corp, Hanover, MD, USA, 0.1 cm precision) and seca electronic scale (5 g precision). If the two length measurements differed by > 0.4 cm, a third measurement was taken. The mean of the two closest length measurements were recorded.

### Fecal collection, transport and storage

A plastic container was distributed to the mothers who were instructed on collection of a stool sample and to take precautions against contamination with soil or dirt. The stool sample was collected on the same day or a day before the study visit. Samples were stored at −20°C. The samples from Yunnan rural infants were transferred frozen to Shanghai.

### Calprotectin assays

The fecal samples were analyzed within three months of collection. FC levels were measured using an EK-CAL ELISA kit (Bühlmann Laboratories AG, Switzerland), following the manufacturer’s instructions. The frozen samples were defrosted at room temperature. The empty polypropylene tube together with the inoculation loop was labeled and weighed. Approximately 50 ~ 100 mg of the fecal sample were transferred into a pre-weighed tube by means of an inoculation loop. The net amount of sample was weighed then broken off the inoculation loop leaving the lower part of the loop in the tube. Extraction buffer (49 × net weight) was added to the tube and the tube was closed before sample homogenization on a multi-tube vortexer with vigorous shaking for 30 min. The homogenate was transferred into a 2 mL polypropylene tube and centrifuged in a microcentrifuge for 5 min at 3000 × g. The supernatant was transferred into a fresh, labeled tube for the ELISA procedure. The lower range procedure (working range 10 ~ 600 μg/g) was chosen to perform the ELISA assay in the first two batches which included 98 samples. The fecal extracts were diluted 1:50 with incubation buffer and the absorbance was read at 450 nm in a microtiter plate reader. Unexpectedly, 29 samples, all but one from the rural infants, read higher than the top calibration. Because no sample material was available for further analysis, these samples could only be quantified as having FC levels > 600 μg/g. Subsequently, the remaining extract was stored and if analytical results exceeded the top calibrator, second fecal extracts were further diluted 1:6 with incubation buffer and assayed again.

### Statistical analyses

(LAZ), weight-for-age (WAZ), and weight-for-length (WLZ) Z-scores were calculated with Anthro (version 3.1) using World Health Organization Child Growth standards. Comparison tests of LAZ, WAZ and WLZ were performed using t-tests. Because FC data were found to have a non-normal distribution, comparison tests of these data were conducted using the non-parametric Mann–Whitney Test. The non-parametric test also accommodated the presence of the 29 data having unknown fecal calprotectin values > 600 μg/g. In cases where the rankings of the unknown data affected the *P*-values of tests, hypothetical FC values that produced the extreme ranking possibilities were used and the resulting *P*-value closest to the significance level was reported. The test of association (contingency) between FC ranges and location was performed with the Fisher Exact Test. Tests were two-sided with a significance level of 0.05.

Simple linear regression analysis of LAZ (as the response variable) and FC was performed on the data from both groups. Because the rural data included a subset having FC values > 600 μg/g lacking precise measurement and the absence of these data would bias the regression results, Monte Carlo simulation was used to account for these data and estimate regression parameter values and confidence intervals. The simulated data were assigned FC values calculated with a function of the frequency distribution of the measured FC data and LAZ values were generated with random Gaussian variation from a line of linear association of LAZ and FC. The line and the magnitude of the variation were estimated from the frequency distribution and mean of the LAZ values of the 28 data, the median of the FC values > 600 μg/g and a slope determined from a series of preliminary simulations. One thousand sampling iterations were performed and in each case the 28 generated values were combined with the 77 accurately measured data and linear regression performed. Mean slope and intercept values and their 95% confidence intervals were calculated from the results.

An estimate of the proportion of the mean negative LAZ for the rural group that was attributable to gut inflammation as indicated by the FC values for that group was calculated as the difference, expressed as a percentage of the mean negative LAZ, between the mean rural LAZ and the LAZ calculated from the urban (normal control) median FC value using the rural regression line. Analyses were accomplished using Graphpad Prism (version 5.04, GraphPad Software, San Diego CA USA) and R (version 2.13, The R Project for Statistical Computing, http://www.r-project.org).

## Results

The general characteristics and anthropometric data of the infants are shown in Table 1. There were 144 infants, 105 from the rural area and 39 from the urban area, 75 M/69 F. The mean age was 6.1 ± 0.2 mo (range: 5 mo 17 d to 6 mo 30 d. The mean values of LAZ, WAZ and WLZ for the rural infants were significantly lower than those for the urban infants (*P*-values < 0.0001, < 0.0001 and 0.0003, respectively). The WLZ scores for males and females were significantly different for both the urban and rural infants (*P =* 0.022 and 0.007, respectively). All rural infants were breast fed; 20 of the urban infants were not receiving any breast milk at the time of the study and 50% received some infant formula. Only a few Xichou infants received formula. All infants in both groups received some complementary foods by the age of four months, including rice, egg yolk and fruit.

**Table 1:** **Characteristics of the study population**^**1**^

	**Rural infants**			**Urban infants**		
	**All** **(*n*** **= 105)**	**Male** **(*n*** **= 55)**	**Female** **(*n*** **= 50)**	**All** **(*n*** **= 39)**	**Male** **(*n*** **= 20)**	**Female** **(*n*** **= 19)**
**Age, mo**	6.0 ± 0.2	6.0 ± 0.2	6.1 ± 0.2	6.2 ± 0.3	6.3 ± 0.3	6.2 ± 0.2
**Birth wt, kg**	3.1 ± 0.4	3.2 ± 0.4	3.0 ± 0.4	3.4 ± 0.5	3.5 ± 0.6	3.3 ± 0.4
**Weight, kg**	7.4 ± 0.9	7.8 ± 0.8	7.0 ± 0.8	8.4 ± 0.9	8.9 ± 0.7	7.9 ± 0.8
**Length, cm**	65.5 ± 2.2	66.1 ± 2.1	64.7 ± 2.1	67.8 ± 2.3	68.6 ± 2.0	67.0 ± 2.3
**LAZ**^**2**^	−0.6 ± 0.9	−0.7 ± 0.9	−0.5 ± 0.9	0.4 ± 0.9	0.4 ± 0.9	0.5 ± 1.0
**WAZ**^**3**^	−0.3 ± 1.0	−0.1 ± 1.0	−0.5 ± 1.0	0.8 ± 0.8	1.0 ± 0.7	0.6 ± 0.8
**WLZ**^**4**^	0.2 ± 1.0	0.4 ± 0.9	−0.1 ± 1.0	0.8 ± 0.8	1.1 ± 0.7	0.6 ± 0.7

^**1**^Data are presented as mean ± SD; ^2^Length-for-age Z-score; ^3^Weight-for-age Z-score; ^4^Weight-for-length Z-score.

The mean values of LAZ, WAZ and WLZ for all subjects with FC values < 350 μg/g, a reported upper cut off during the first year of life [17], were −0.0007, 0.315 and 0.517 respectively, compared to −0.753, -0.427 and 0.136 for subjects with FC values > 350 μg/g. In each case the difference was significant (*P* < 0.0001, < 0.0001 and 0.021 respectively).

The median FC level was 281.5 μg/g (known range: 24.5 ~ 1267.9 μg/g) in all infants. The median FC level was significantly higher in rural infants (420.9 μg/g, known range: 24.5-1268 μg/g) than in urban infants (140.1 μg/g, known range 26.2-1258 μg/g) (*P* < 0.0001). The median FC levels were not different for the 75 males (243.8 μg/g, known range: 26.2-1034 μg/g) and 69 females (300.9 μg/g, known range: 24.5-1268 μg/g). Fifty-six percent and 10% of rural and urban measurements, respectively, were > 350 μg/g. This association of FC range and location was significant (*P* < 0.0001).

**Figure 1:**
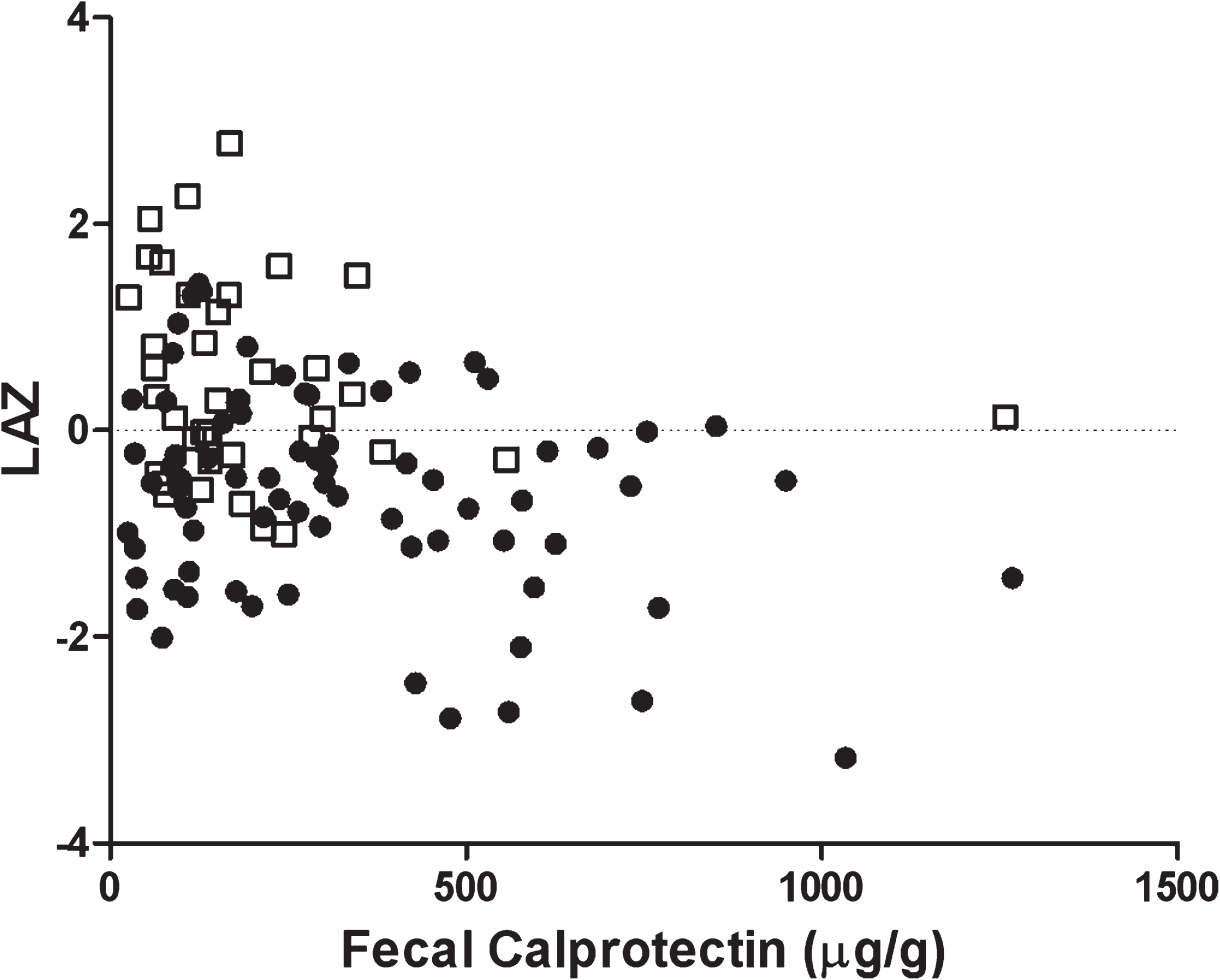
**Length-for-age Z-scores as a function of fecal calprotectin.** Symbols denote rural and urban infants.

Simple linear regression of LAZ vs. FC for the urban infants showed that the relationship was not significant (Figure 1, *P* = 0.34). Addition of an estimate for the single datum having an unknown value > 600 μg/g had negligible effect on these results. Regression analysis of the rural data using Monte Carlo simulation indicated that the relationship of LAZ and FC was significant (*P* = 0.014); the estimate for the slope was −0.00061 with a 95% confidence interval of −0.00013 to −0.00109. The slope confidence interval indicated that LAZ and FC had a significant inverse relationship; more specifically, a 100 μg/g increase in FC was associated with a 0.06 decrease in LAZ. Examination of the residuals indicated no apparent departures from the regression assumptions. There was no correlation between birth weight and FC.

The proportion of the low mean LAZ for the rural group (−0.6) attributable to high FC was estimated to be 31% on average.

## Discussion

It should be emphasized that this was an exploratory study included as a last minute addition to the baseline data for the parent study in Xichou followed by recognition of the potential interest in comparing with the upper-middle income urban children. Sample sizes were dictated by timelines rather than power testing. Statistics were complicated by some values for calprotectin in Xichou infants exceeding expectations and, therefore, the assay range established for the first batch. Hence, this should be regarded as a pilot observational study and our discussion viewed with this in mind.

In our model, the higher median FCC of the rural vs urban (control) infants accounted for one/third of the linear growth deficit. The results of this project suggest that the measurement of calprotectin concentrations in small random fecal samples provides a simple, non-invasive strategy for the detection of gut inflammation in apparently normal infants and a measure of the severity of this inflammation. It has been hypothesized that in these circumstances there is translocation of immunogenic luminal macromolecules across a compromised gut mucosa leading to stimulation of systemic immuno/inflammatory processes and subsequent linear growth impairment [2]. The estimated contribution to linear growth faltering is less than that estimated for rural Gambian infants [2]. In contrast, FC levels were not elevated in urban children aged 0–12 y in urban Kampala [18] which suggests either a more favorable environment or a protective effect associated with increasing age. The median FC value of 140 μg/g for the urban infants was a little higher than that for the only other reported data for normal infants) at the age of 6 mo [19]. Our urban data, therefore, provided a quite conservative control for comparison with the rural infant data. It is noted that fecal CP levels were subsequently found to be modestly higher for age in hospitalized young children in Kampala who were HIV-infected, highly active anti-retroviral-naïve Ugandan children. Children with advanced disease had significantly higher FC levels and this also applied to older children with diarrhea [20].

Consistent with previous studies [19,21-26], the FC levels observed in healthy 6 mo infants in the current study were higher than in healthy adults and children. The high FC levels in infants may reflect the increased trans-epithelial migration of neutrophil granulocytes and/or macrophages into the intestinal lumen, which could be due to the greater intestinal permeability of young infants and may reflect an increase in leukocyte migration through the gut mucosa, as part of the development of the gut-associated lymphoid tissue which is particular to this period of life [27].

As ages of urban and rural infants were the same, age-related differences did not account for the rural versus urban observed differences in FC. Breast feeding has in some, but not all, reports have been associated with higher FC levels in younger infants [24,27]. However, this difference does not account for the high FC value in the rural group at 6 months nor for the association between FC and linear growth in the rural group. Calprotectin levels are already used and documented extensively in the monitoring of the extent of inflammation of the gut in inflammatory bowel disease [28-30]. However, the application of this assay in assessing the integrity of the gut mucosa in infants with growth failure requires further studies prospectively designed to evaluate this as well as other recently proposed inflammatory markers that can be assayed in the feces. FC has the special advantage in infants and young children of not requiring venipuncture. The simplicity of collection and storage add further advantages.

## Conclusion

These results are consistent with the hypothesis that chronic sub-clinical intestinal inflammation is a greater problem in relatively poor rural infants in contrast to those living in a relatively affluent urban environment. Moreover, this apparent chronic low-grade inflammation is associated with impairment of linear growth.

## Abbreviations

FC: Fecal calprotectin; LAZ: Length-for-Age Z-score; WAZ: Weight-for-Age Z-score; WLZ: Weight-for-Length Z-score.

## Competing interests

None of the authors have any competing interests to disclose.

## Author contributions

KMH, NFK, and XYS conceived of and designed the research project. XYS had oversight responsibility for the studies undertaken in China including the human studies in Yunnan province and the fecal calprotectin assays in Shanhgai. JRL and YQH were leaders in outcome assessment and data collection, processing. LVM assisted with the data analysis. JRL, KMH, LVM, XYS, NFK, and JEW prepared the manuscript and KMH, XYS and LVM had primary responsibility for final content. All authors have read and approved the final manuscript.

## Pre-publication history

The pre-publication history for this paper can be accessed here:

http://www.biomedcentral.com/1471-2431/12/129/prepub
